# Antibacterial, Antioxidant Activity of Ethanolic Plant Extracts of Some* Convolvulus* Species and Their DART-ToF-MS Profiling

**DOI:** 10.1155/2017/5694305

**Published:** 2017-11-26

**Authors:** Asma'a Al-Rifai, Ahmad Aqel, Tarfah Al-Warhi, Saikh M. Wabaidur, Zeid A. Al-Othman, A. Yacine Badjah-Hadj-Ahmed

**Affiliations:** ^1^Department of Chemistry, College of Science, Princess Nourah bint Abdulrahman University, Riyadh, Saudi Arabia; ^2^Department of Chemistry, College of Science, King Saud University, Riyadh, Saudi Arabia

## Abstract

*Convolvulus austroaegyptiacus* Abdallah & Sa'ad* (CA)* and* Convolvulus pilosellifolius* Desr.* (CP) *are commonly used in the Saudi Arabia folk medicine. They are potent in treating the ulcers and skin diseases. The lack of information about their biological activities led us to investigate the possible biological activities by determination of antibacterial and antioxidant activities of total ethanolic extracts and various fractions. Total flavonoid contents of the plants were determined by colorimetric method while total phenols were determined by using Folin-Ciocalteu method.* In vitro* antibacterial activity was studied against* E. coli*,* P. aeruginosa*, and* B. subtilis*, and the total antioxidant capacity was evaluated by radical scavenging method. IC_50_ were found to be 21.81, 17.62, and 3.31 *μ*g/mL for* CA*,* CP*, and vitamin C, respectively, while the lowest MIC value of 0.25 mg/mL was recorded with* CP* extract against* B. subtilis.* Around 21 compounds are tentatively elucidated from both plants using rapid, simple, and high-resolution analytical technique for chemical profiling of natural compounds by direct analysis in real-time of flight-mass spectrometry, of which 17 were not isolated or reported previously.

## 1. Introduction

Traditional medicine from plant extracts has proved to be clinically effective and relatively less toxic than the existing drugs [1]. Successful determination of biologically active compounds from plant material is largely dependent on the type of solvent used in the extraction procedure [2]. Many solvents have been used to extract active materials from plants such as alcohols (ethanol or methanol), diethyl ether, chloroform, ethyl acetate,* n*-butanol, and water [3].

Phytochemicals (secondary metabolites) are bioactive chemicals of plant origin. They are naturally synthesized in all parts of the plant body: bark, leaves, stems, roots, flowers, fruits, seeds, and so on [2]. They have been recognized as the basis for traditional herbal medicine practiced in the past and now [4]. All plant parts are usually screened for phytochemicals that may be present; the presence of a phytochemical of interest may lead to its further isolation, purification, and characterization. Then it can be used as the basis for a new pharmaceutical product.

Free radicals which are delivered as a consequence of typical biochemical responses in the body are involved in cancer, ischemic heart disease, inflammation, diabetes, aging, atherosclerosis, immunosuppression, and neurodegenerative disorders [5]. The human body has characteristic barrier system to counter free radical as proteins, for example, catalase, superoxide dismutase, and glutathione peroxidase. Selenium, vitamin C, *β*-carotene, vitamin E, lycopene, lutein, and different carotenoids have been utilized as supplementary antioxidants. Hence, the secondary metabolites of the plant as flavonoids and terpenoids act an important role in the defense against free radicals [6].

Recently, researchers have focused on increasing human infections caused by bacteria and fungi. Medicinal plants represent a rich source of antimicrobial agents. Because microorganisms have developed resistance to many antibiotics [7], the use of plant extracts and their isolated compounds as a resistant against microorganisms has been increased [8].

Antibacterial activity of different plant extracts was documented in the literature using various organic solvents such as ethanol, methanol, and petroleum ether. Muslim and his coauthor [9] studied the antibacterial activity of* Convolvulus arvensis* and the results demonstrated a promising effect. A significant cytotoxic activity for essential oil of* Convolvulus althaeoides* L. was reported [10]. Our search was carried out with the total extract (ethanol) and other solvent soluble fractions (diethyl ether, chloroform, ethyl acetate, and* n*-butanol) to found out the ability of traditional medicinal plants* CA* and* CP* collected from Riyadh, KSA, as an antibacterial agents.

Convolvulaceae (bindweed family) is one of the largest plant families; it includes mostly twining herbs or shrubs, comprising about 85 genera and 2800 species that are further characterized by having the flowers solitary or in terminal [11]. Convolvulaceae family is well known and distinctive as a whole; however its division into species and genera might be difficult. In order to make the taxonomic relationship clear, seed morphology has been studied [12]. This family contains a large number of important plants which are used in treatment of various diseases such as headache, constipation, rheumatism, diabetes, and skin infection [13]. Members of this family mainly contain phenolics, tropane alkaloids, flavonoids, and coumarins [14].

*Convolvulus* is the largest genus of family Convolvulaceae; it contains 250 species present as herbs, trees, or shrubs [15]. Many* Convolvulus* species are valuable ornamentals, medicinal and food crops [16]. They possess cytotoxic [17], antioxidant, anti-inflammatory, antiasthma, antijaundice [18], anticancer, and antiulcer activities [19]. In addition, they can be used in treatment of coughs and asthma [13]. They contain many phytochemical groups such as phenolics, tropane alkaloids, sterols, resins, and sugars [14].

*Convolvulus austroaegyptiacus* Abdallah & Sa'ad* (CA) *and* Convolvulus pilosellifolius* Desr.* (CP)* are some of* Convolvulus *species. These plants are creeping or twining herbs or shrubs of various appearances and habits. Leaves are undivided or lobed, if parted mostly 1 of glabrous calyx, relatively big, not crowded, funnel-shaped, with entire, 5-angled or 5-plaited limb, and no regular colored stripes like those of Ipomoea [15]. These plants are mainly used in Saudi Arabia as folk medicine for treatment of ulcer diseases. Our research group studied the antiulcerogenic activity of* CA* and the results showed a promising activity [20]. Although the antiulcerogenic activity of* CP* is still under study, two compounds (quercetin and kaempferol) were characterized in its composition using HPLC [21].

Direct analysis in real-time of flight-mass spectrometry (DART) is a relatively recent ion source. Its atmospheric pressure ion source instantaneously ionizes any physical state samples (gases, liquids, or solids) in their native state under ambient conditions [22]. The power of DART ion source appears when it is combined with high-resolution mass analyzer as it gives exact molecular weight of ionized compounds from the samples and therefore provides matching molecular formula [23]. Recently, DART-ToF-MS is a strong candidate as a rapid and powerful technique for profiling of several types of samples including the major constituents of total plant species with high resolution mass number directly in real time. Many qualitative analyses of different organic compounds including phytochemicals, metabolites, pharmaceuticals, and synthetic organic molecules have been approved by DART-MS [24]. Since sample preparation or vacuum ionization steps can be omitted in DART-MS analysis, high throughput fingerprinting study of natural samples is possible and this feature is considered one of the most advantageous characteristics of DART ion source [23]. In addition to that, DART ion source ionizes moderately polar to highly nonpolar compounds [25]. Therefore, phenolic compounds are easily ionized and suitable for detection.

**Figure 1:**
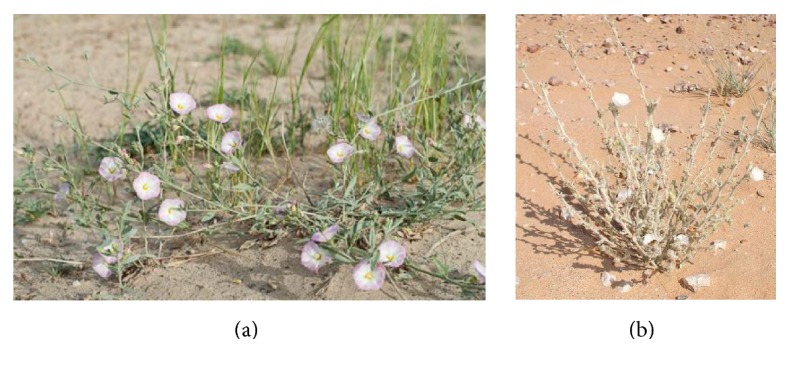
The two plants under investigation were (a)* CA* and (b)* CP* plants.

To our best knowledge,* CA* and* CP* plants are used in folk medicine for treatment of various diseases; however, they have never been studied as antioxidant and antibacterial. In addition, this is the first study using a unique DART-ToF-MS for identification of chemical constituents of the two plants in which new compounds have been identified for the first time in these plants. Consequently, this work has been intended to explore the phytochemical screening and to assess the flavonoids content, total phenol, antimicrobial, antioxidant potentials and DART profiling of the total extracts and different fractions obtained from the aerial parts of* CA *and* CP*.  Figure 1 shows the plants under investigation.

## 2. Materials and Methods 

### 2.1. General

Polyethylene glycol (PEG) with average relative molecular weight of 600 was purchased from Sigma-Aldrich, (Steinheim, Germany). All other solvents and chemicals used were purchased at analytical grade from Merck (Darmstadt, Germany). UV spectra were recorded on UV-1800 Shimadzu spectrophotometer (Tokyo, Japan) (*λ*_max_ in nm).

### 2.2. Collection and Preparation of Samples

Aerial parts of* CA *and* CP *were collected during flowering stage from the Alrawda, Riyadh district, Saudi Arabia. Samples of the aerial parts were air dried in shade, reduced to fine powder, and kept for total phenols, flavonoids content, and phytochemical investigation.

### 2.3. Extraction and Fractionation Procedure

100 g of air dried powder from each plant under investigation (*CA *and* CP*) was separately extracted by refluxing in 200 mL ethanol 70% three times for 3 hours, filtered off, and concentered using rotary evaporator to obtain 50 g from each plants. The residue obtained from each plant was separately suspended in 200 mL water followed by filtering over a piece of cotton. The filtered aqueous layers were successively fractionated using several solvents such as diethyl ether, chloroform, ethyl acetate, and* n*-butanol, and the extracts were dried using anhydrous sodium sulfate, followed by concentrations to obtain 5, 3, 10, and 30 g for diethyl ether, chloroform, ethyl acetate, and* n*-butanol, respectively, from* CA *and 3, 2, 9 and 31 g for diethyl ether, chloroform, ethyl acetate, and* n*-butanol, respectively, from* CP.*

### 2.4. Preliminary Phytochemical Screening

Preliminary phytochemical screening was carried out for the ethanolic plant extracts and the four fractions from each plant (diethyl ether, chloroform, ethyl acetate, and* n*-butanol) for their usual plant secondary metabolites using the standard methods [26]. The screening was performed for coumarins, flavonoids, saponins, tannins, triterpenes/steroids, alkaloids, phenolic compounds, and anthraquinones. The precipitate formation or the color intensity was used as analytical responses to these tests.

### 2.5. Determination of Total Phenolic Compounds Contents

Total phenolic content in the plant ethanolic extract and fractions was determined using spectrophotometric method [27]. Briefly, 0.5 mL of Folin-Ciocalteu's reagent and 1 mg from total extract and fractions was mixed separately with 10 mL of distilled water for 3 min, followed by adding of 1 mL of saturated Na_2_CO_3_; then the volume was made up to 25 mL. The samples were kept for 1 h in the dark place and the absorbance was measured at 750 nm wavelength against a blank. The samples were prepared in triplicate for each analysis and the mean value of absorbance was obtained. Different concentrations of gallic acid (5, 10, 20, 40, 60, 80, 100, and 150 mg mL^−1^) were prepared to obtain a calibration curve. The total phenolic content was expressed in milligrams of gallic acid equivalent per gram of extract [28].

### 2.6. Determination of Total Flavonoids Contents

Total flavonoids content was determined by a colorimetric method. 0.25 mg from total extract and fractions was separately diluted with 5 mL of distilled water in a 10 mL measuring flask. Then 0.3 mL of a 5% NaNO_2_ solution was added to the mixture. The test tubes were left in the dark for 6 min, then 0.6 mL of a 10% AlCl_3_·6H_2_O solution was added, and the tubes were returned to the dark place for complete reaction. Consequently, 2 mL of 1 M NaOH was added to the mixture, and the total volume was made up to 10 mL with distilled water. After mixing of the solution, the absorbance was measured at 510 nm wavelength. The samples were prepared in triplicate for each analysis and the mean value of absorbance was obtained. The flavonoid content was calculated using a quercetin calibration curve and the results were expressed as mg of quercetin equivalent per gram of extract [29].

### 2.7. Evaluation of Antioxidant Activity

The antioxidant activity of the two plant crude extracts against 2,2-diphenyl-1-picrylhydrazyl (DPPH) radical was determined by UV spectrophotometry at 518 nm. The activity was measured according to the method previously described [30]. Different concentrations of the plant extracts were prepared using analytical methanol (1, 3, 7, 10, 20, 30, 40, 50, 80, and 100 *μ*g mL^−1^). Vitamin C was used as an antioxidant standard. 1 mL from each extract and 3 mL of methanol were mixed by 0.5 mL of 1.0 mM DPPH in methanol and allowed to react at room temperature for 30 minutes. Same amount of methanol and DPPH was mixed to prepare the blank solution. The samples were prepared in triplicate for each analysis and the mean value of absorbance was obtained. The radical scavenging activity was calculated using the following formula:

(1) $$\begin{array}{c} \%\;\;inhibition=\frac{Ab-Aa}{Ab}\times 100 \end{array}$$

in which *Ab* is absorption of the blank sample and *Aa* is absorption of the extract.

Extract concentration providing 50% inhibition (IC_50_) was calculated from the plot of inhibition percentage against extract concentration.

### 2.8. Evaluation of Antibacterial Activity

The crude plant extracts and their successive fractions were subjected to antibacterial evaluation. Susceptibility of the bacterial strains to the two plant extracts and fractions was investigated using disk diffusion method [31]. The concentration of the cell suspension was adjusted to the 2.0 McFarland standard [30] and 50 *μ*L of each microorganism's suspension was spread on a Mueller-Hinton agar plate. Filter paper disks of 5 mm diameter impregnated with 5 mg of each extract and fraction (50 *μ*L of stock solutions) were placed onto the surface of the agar and incubated at 37°C for 24 h. The antibacterial activity of the plant extracts and fractions was tested against three microorganisms,* B. subtilis, P*.* aeruginosa*, and* E. coli.* Doxycycline (20 *μ*g mL^−1^) was used as positive control and ethanol solvent was used as negative control. The diameters of the inhibition zones were measured to the nearest millimeter.

### 2.9. Determination of Minimum Inhibitory Concentration (MIC)

MIC represents the lowest extract concentration which prevents the microorganism visible growth. It was carried out for the bacterial strains to the ethanolic extracts of* CA* and* CP*. A 100 *μ*L of the inoculum was spread onto 20 mL Mueller–Hinton broth (MHB) supplemented with the extracts at concentrations ranging from (0.0–100 mg mL^−1^) in Petri dishes, with 5% dimethyl sulfoxide (DMSO). These serially cultures were then incubated at 37°C for 48 h. 10% DMSO was used as a control [31].

### 2.10. DART-ToF-MS Conditions

High-resolution mass measurements were carried out using a Jeol the AccuTOF LC-plus JMS-T100 LP atmospheric pressure ionization ToF-MS (Jeol, Tokyo, Japan) equipped with a DART ion source (IonSense, Saugus, MA, USA). The mass spectrometer was operated in +ve-ion mode. The orifice 1 and 2 potentials were set to 20 and 5 V. The ring lens potential was set to 13 V. Orifice 1 was set to a temperature of 80°C. The RF ion guide potential was 300 V. The DART ion source was operated with He gas at 4.0 L min^−1^ flow. The gas heater was set to 200°C. The potential on the discharge needle electrode of the DART source was set to 3.0 kV; perforated and grid electrode potentials were at 100 and 250 V, respectively.

Data acquisition was monitored in the range of 10 to 1000* m/z* at acquisition rate 5 spectra min^−1^. The distance between the DART gun exit and mass spectrometer inlet was 10 mm. In order to perform mass drift compensation for accurate mass measurements, a PEG with 200 *μ*g mL^−1^ solution in methanol was applied before each analysis run. The elemental composition has been determined on selected peaks using the MassCentre software, version 1.3.m.

### 2.11. Statistical Analysis

All experiments were done in triplicate and the data were expressed as the mean ± SD. IC_50_ values were determined by interpolation.

## 3. Results and Discussion

### 3.1. Phytochemical Screening

The two plant extracts were defatted with water followed by extracted with different organic solvents according to increasing the polarities; diethyl ether, chloroform, ethyl acetate, and* n*-butanol. Results obtained from qualitative analysis of phytochemicals of the total ethanolic extracts and their successive fractions of both plants under investigation (*CA *and* CP*) are presented in Table 1.

Phytochemical analysis revealed the presence of coumarins, steroids, flavonoids, saponins, tannins, triterpenoids, carbohydrates, amino acids, and proteins, while both alkaloids and anthraquinones were absent in the crude extracts for both plants. Only steroids and triterpenoids were present in both diethyl ether and chloroform fractions. The chemical contents in the different fractions are considered as secondary metabolite components which are known to be biologically active ingredients. They are directly responsible for different activities such as antimicrobial, antioxidant, antifungal, and anticancer [32]. Normally these secondary metabolites components were isolated from the polar extract [33]. The most important bioactive compounds, which are flavonoids, were found in ethanol (total extract) and* n*-butanol fraction for both plants under investigation, while traces of flavonoids were present in ethyl acetate fraction. Many researchers reported that flavonoids and phenolic compounds in plants proved to possess multiple biological effects including antioxidant, anti-inflammatory, antimicrobial, antiangiogenic, anticancer, and antiallergic activities. In general, phenolic compounds and their derivatives are also considered as primary antioxidants or free radical scavengers [34].

### 3.2. Total Phenolic Compounds and Total Flavonoid Contents

The total phenolic contents of the ethanolic extract of* CA *and* CP *and their successive fractions were determined using the Folin-Ciocalteu reagent in comparison with standard gallic acid, and the result was expressed in terms of mg gallic acid g^−1^ extract. The results for both plants in Table 2 indicated that the total phenolic of the total extract (ethanolic extract) for* CA *(125.44 mg g^−1^) is lower than for* CP *(129.97 mg g^−1^). Total phenolic in ethyl acetate fraction of both plants (146.83 and 158.14 mg g^−1^ for* CA *and* CP*, resp.) was higher than those which found with* n*-butanol fraction (92.00 and 95.05 mg g^−1^ for* CA *and* CP*, resp.) followed by chloroform then diethyl ether fractions.

Colorimetric estimation of the total flavonoids of ethanolic extract of both plants and their successive fractions calculated based on quercetin proved that the total flavonoid contents of the total extract (ethanolic extract) for* CA *(91.61 mg g^−1^) was lower than that of* CP *(92.58 mg g^−1^). The total flavonoids in ethyl acetate fraction of both plants (123.88 and 125.92 mg g^−1^ for* CA *and* CP*, resp.) were higher than those of* n*-butanol fraction (45.40 and 46.37 mg g^−1^* CA *and* CP*, resp.) followed by chloroform and diethyl ether fractions.

According to the total phenolic and total flavonoids assay, the obtained results proved that* CP* extract has the highest total phenol and flavonoid contents. This demonstrated that the phenolic compounds of these plants might consist mainly of flavonoids, which are mainly in the form of glycosides, as they are more concentrated in the polar solvents, which is more effective in extracting phenolic compounds from plant materials than the less polar solvents [35]. Previous studies recorded that phenolic compounds including flavonoids are associated with strong antioxidant activity and they possess healthy benefits [36].

### 3.3. Antioxidant Activity

The antioxidant activity of ethanolic extract of* CA* and* CP* was measured by the ability to scavenge DPPH free radicals comparing with vitamin C. The scavenging effects of both plant extracts and the standard on the DPPH radical were expressed as half maximal inhibitory concentration (IC_50_) values; the results are reported in Table 3. Lower IC_50_ value reflects higher DPPH radical scavenging activity. According to the results obtained, the ethanolic extract of* CP* showed significant DPPH activity with the IC_50_ value of 17.62 *μ*g mL^−1^, while IC_50_ of vitamin C as standard was 3.31 *μ*g mL^−1^.

Different experiments have been performed to recognize the property of plant extracts to scavenge the free radicals [37]. DPPH is a free radical compound which has scavenging ability for antioxidants samples and shows good absorbance at 517 nm [38]. Vitamin C is usually used as a standard antioxidant and it has a strong DPPH scavenging property [39]. Several studies demonstrated a linear correlation between antioxidant activity and phenolic content of plant extracts [40]. Our findings were not an exceptional case, as they indicated that* CP *extract consists of high quantity of phenolic compounds and showed promising antioxidant activity with 17.62 *μ*g mL^−1^ IC_50_ value.

### 3.4. Antibacterial Activity

To our knowledge, no previous publications have reported the antibacterial activity of our plants. Therefore, antibacterial activity of total extract and successive fractions of* CA* and* CP* against the tested bacteria strains was assessed by the presence and absence of inhibition zones using disk diffusion method. The inhibition zone produced by the crude extracts and fractions for both plants on different bacterial strains was between 4 mm and 20 mm. The antimicrobial studies revealed that the ethanolic extract and fractions of* CP* showed inhibitory effects on* B. subtilis*,* P. aeruginosa*, and* E. coli*, while the crude extract and fractions of* CA* exhibited less inhibitory effects on these microorganisms as shown in Table 4. The MIC value was lowest for the ethanolic extract of* CP* (0.25 mg mL^−1^) against* B. subtilis* followed by the ethanolic extract of* CA* (0.78 mg mL^−1^) against* B. subtilis* (Table 5).

Many previous studies revealed that the plants play an important role in development of new therapeutic sources. The first step in order to achieve this target is the* in vitro* antibacterial activity test [41]. Reviewing the literature, the presence of the secondary metabolites in the plant extract like saponins, tannins, flavonoids, coumarin, phenol, and triterpenoids has a promising activity against pathogens and helps the antibacterial activities of plants [42]. According to previous study, the ability of plant extracts against bacteria depends on the solubility of the bioactive constituents [43]. Our results clarified that the crude extract of* CP* proved its efficiency to be used as source for antibacterial compounds compared with some standard antibiotics due to its inhibitory effects on* B. subtilis*,* P. aeruginosa*, and* E. coli* with lowest MIC value (0.25 mg mL^−1^) against* B. subtilis*.

### 3.5. DART-ToF-MS of the Plants Extract

**Figure 2:**
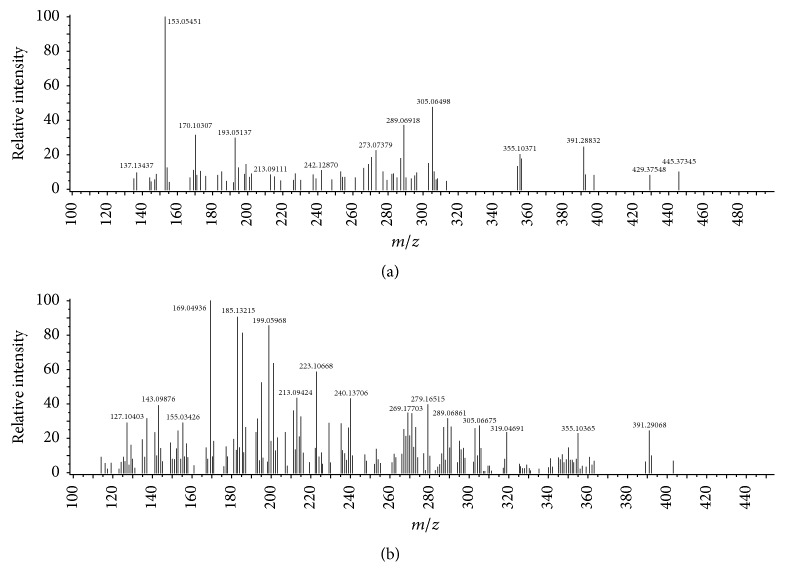
DART mass spectra of (a)* CA* and (b)* CP* plants.

Spectrometry analysis is one of the most powerful analytical methods available for exact structural identification of organic compounds. In this study, DART-ToF-MS profiles of the total* CA* and* CP* plants extract were obtained. Both plants were subjected to DART-ToF-MS analysis under the same conditions. As shown in Figure 2 and reported in Table 6, peaks corresponding to the molecular species of phellandrenes (*m/z* 137), p-hydroxyphenylacetic acid (*m/z* 153), scopoletin (*m/z* 193), ferulic acid (*m/z* 195), syringic acid (*m/z* 199), pinosylvin (*m/z* 213), apigenin/galangin (*m/z* 271), naringenin (*m/z* 273), kaempferol/luteolin/fisetin (*m/z* 287), eriodictyol/aromadendrin (*m/z* 289), quercetin (*m/z* 303), taxifolin (*m/z* 305), and scopolin (*m/z* 355) were observed in the DART mass spectra in both plants. All selected peaks of the major components were on the type of protonated molecular ions [M + H]^+^ related to the highest peak intensity of the same compound. In addition, the unsaturated degree of each compound is reported in Table 6.

Furthermore, three compounds, protocatechuic acid/gentisic acid, vanillic acid, and myricetin corresponding to* m/z* 155, 169, and 319, respectively, have been observed in* CP* plant but not detected in* CA* plant.

Since its soft ionization technique, DART ion source provides relatively simple mass spectra with minimal fragmentations, consisting of the major constituents of the protonated molecules in the case of positive ion mode. All the results prove that DART-ToF-MS is an appropriate method to differentiate between the two studied plants and for rapid identification of their major components, which mainly consist of flavonoids and phenols as mentioned in the phytochemical screening section.

On the other hand, the unsaturated degree of each compound helps to predict the chemical structures by determination how many double bond and rings are present in the compound, which also confirms the expected flavonoids and phenols compounds. It should be noted that some compounds such as apigenin and galangin have the same molecular formula and exact mass. Therefore, they cannot be distinguished on the basis of high-resolution mass spectrometry using DART-ToF-MS technique.

## 4. Conclusion

We demonstrate that DART-ToF-MS combined by multivariate analysis allows for rapid screening and metabolic characterization of different compounds from plant extracts without complex metabolic preparation steps such as phenol and flavonoid compounds. With this method, 21 phenol and flavonoid compounds are tentatively detected and identified in* CA* and* CP*; 17 of them are reported from these plants for the first time. The obtained results confirmed that DART-ToF-MS data were specific and valuable since it can directly ionize various organic molecules. Therefore, it would be a technique of choice for simple and rapid screening of natural products and a wide variety of materials with very simple or no sample preparation. The newly discovered phenol and flavonoid compounds expand our understanding on the chemical constituents of* CA* and* CP* and can be targets for further phytochemical studies. The literature survey indicated that there is no research that has been carried out on antioxidant potent and antibacterial activity of these plant species; therefore, this study can be a guideline for further biological activities investigations.

## Figures and Tables

**Table 1:** Phytochemical screening of ethanolic extract (total extract) and fractions from *CA* and *CP*.

Group	*CA*					*CP*				
	Diethyl ether	CHCl_3_	EtOAc	*n*-Butanol	Total extract	Diethyl ether	CHCl_3_	EtOAc	*n*-Butanol	Total extract
Alkaloids	−	−	−	−	−	−	−	−	−	−
Flavonoids	−	−	±	+	+	−	−	±	+	+
Saponins	−	−	+	+	+	−	−	+	+	+
Steroids	+	+	−	−	+	+	+	−	−	+
Tannins	−	−	−	+	+	−	−	−	+	+
Triterpenoids	+	+	−	−	+	+	+	−	−	+
Carbohydrate	−	−	±	+	+	−	−	±	+	+
Amino acids & proteins	−	−	+	+	+	−	−	+	+	+
Anthraquinones	−	−	−	−	−	−	−	−	−	−
Coumarins	−	−	+	+	+	−	−	+	+	+

+: present, −: absent, ±: trace, CHCl_3_: chloroform, and EtOAc: ethyl acetate.

**Table 2:** Total phenols (mg gallic acid/g extract) and total flavonoids (mg quercetin/g extract) of *CA* and *CP.*

Plant extract	*CA*		*CP*	
	Total phenols	Total flavonoids	Total phenols	Total flavonoids
Diethyl ether	30.31 ± 0.053	9.15 ± 0.026	27.70 ± 0.020	8.14 ± 0.026
Chloroform	39.00 ± 0.182	20.23 ± 0.026	45.09 ± 0.061	22.33 ± 0.026
Ethyl acetate	146.83 ± 0.089	123.88 ± 0.026	158.14 ± 0.078	125.92 ± 0.035
*n*-Butanol	92.00 ± 0.178	45.40 ± 0.020	95.05 ± 0.046	46.37 ± 0.036
Ethanolic extract	125.44 ± 0.044	91.61 ± 0.062	129.97 ± 0.087	92.58 ± 0.036

Values are mean ± SD of 3 replicates.

**Table 3:** Percentage of inhibition of DPPH and IC_50_ for ethanolic extract of two plants at different concentrations (*μ*g/mL) compared with vitamin C.

Concentration (*μ*g/mL)	% inhibition by plant		% inhibition by vitamin C
	*CA*	*CP*	
1	27.22 ± 0.75	31.84 ± 1.07	43.03 ± 1.10
3	35.90 ± 0.63	39.56 ± 1.10	45.78 ± 0.98
7	38.87 ± 1.51	41.09 ± 0.92	51.32 ± 1.11
10	41.58 ± 1.38	43.18 ± 1.26	56.38 ± 1.21
20	59.20 ± 0.61	60.37 ± 1.58	65.89 ± 1.43
30	63.91 ± 1.23	64.58 ± 1.42	69.40 ± 0.89
40	65.56 ± 1.14	66.21 ± 0.85	71.35 ± 1.32
50	67.11 ± 1.53	69.67 ± 1.45	76.71 ± 1.04
80	83.34 ± 1.19	87.48 ± 1.12	99.54 ± 1.22
100	86.11 ± 1.15	90.17 ± 0.96	100.96 ± 1.06
IC_50_ (*μ*g/mL)	21.81	17.62	3.31

Values are mean ± SD of 3 replicates.

**Table 4:** Antibacterial activity of *CA *and* CP* extracts and fractions.

Plant	Inhibition zone (mm)					Microorganisms tested	Doxycycline (control)
	EtOH (crude)	Diethyl ether	CHCl_3_	EtOAc	*n*-Butanol		
*CA*	7	6	8	6.3	4	*B. subtilis*	13
	16	11	12	10	4.3	*P*. *aeruginosa*	18
	7	6.3	7	9	5	*E. coli*	10

*CP*	9	6.2	10	8.2	5.7	*B. subtilis*	12
	20	12.7	14	16.1	8.9	*P*. *aeruginosa*	20
	9.8	6.5	8	10	7.7	*E. coli*	11

IZ: inhibition zone, EtOH: ethanol, CHCl_3_: chloroform, and EtOAc: ethyl acetate.

**Table 5:** MIC against different strains of bacteria for ethanolic extracts of *CA *and* CP.*

Plant	MIC (mg/mL)	Bacterial strains
*CA*	0.78	*B. subtilis*
	1.54	*P*. *aeruginosa*
	1.20	*E. coli*

*CP*	0.25	*B. subtilis*
	1.06	*P*. *aeruginosa*
	0.93	*E. coli*

**Table 6:** Exact mass data from the DART mass spectra of *CA* and *CP* plant constituents.

Plant	Experimental mass (*m/z*)	Calculated mass (*m/z*)	Mass error (mmu)	Formula	Remarks	Unsaturation degree
*CA*	137.13437	137.13303	1.34	C_10_H_17_	Phellandrenes	2.5
	153.05451	153.05516	−0.65	C_8_H_9_O_3_	p-Hydroxyphenylacetic acid	4.5
	193.05137	193.05007	1.30	C_10_H_9_O_4_	Scopoletin	6.5
	195.06742	195.06572	1.70	C_10_H_11_O_4_	Ferulic acid	5.5
	199.05796	199.06063	−2.67	C_9_H_11_O_5_	Syringic acid	4.5
	213.09111	213.09155	−0.44	C_14_H_13_O_2_	Pinosylvin	8.5
	271.06318	271.06063	2.55	C_15_H_11_O_5_	Apigenin/galangin	10.5
	273.07379	273.07628	−2.49	C_15_H_13_O_5_	Naringenin	9.5
	287.05627	287.05554	0.73	C_15_H_11_O_6_	Kaempferol/luteolin/fisetin	10.5
	289.06918	289.07119	−2.01	C_15_H_13_O_6_	Eriodictyol/aromadendrin	9.5
	303.05202	303.05045	1.57	C_15_H_11_O_7_	Quercetin	10.5
	305.06498	305.06610	−1.12	C_15_H_13_O_7_	Taxifolin	9.5
	355.10371	355.10287	0.84	C_16_H_19_O_9_	Scopolin	7.5

*CP*	137.13172	137.13303	−1.31	C_10_H_17_	Phellandrenes	2.5
	153.05573	153.05516	0.57	C_8_H_9_O_3_	p-Hydroxyphenylacetic acid	4.5
	155.03426	155.03442	−0.16	C_7_H_7_O_4_	Protocatechuic acid/ gentisic acid	4.5
	169.04936	169.05007	−0.71	C_8_H_9_O_4_	Vanillic acid	4.5
	193.05084	193.05007	0.77	C_10_H_9_O_4_	Scopoletin	6.5
	195.06620	195.06572	0.48	C_10_H_11_O_4_	Ferulic acid	5.5
	199.05968	199.06063	−0.95	C_9_H_11_O_5_	Syringic acid	4.5
	213.09424	213.09155	2.69	C_14_H_13_O_2_	Pinosylvin	8.5
	271.06165	271.06063	1.02	C_15_H_11_O_5_	Apigenin/galangin	10.5
	273.07482	273.07628	−1.46	C_15_H_13_O_5_	Naringenin	9.5
	287.05517	287.05554	−0.37	C_15_H_11_O_6_	Kaempferol/luteolin/fisetin	10.5
	289.06861	289.07119	−2.58	C_15_H_13_O_6_	Eriodictyol/aromadendrin	9.5
	303.05172	303.05045	1.27	C_15_H_11_O_7_	Quercetin	10.5
	305.06675	305.06610	0.65	C_15_H_13_O_7_	Taxifolin	9.5
	319.04691	319.04536	1.55	C_15_H_11_O_8_	Myricetin	11
	355.10365	355.10287	0.78	C_16_H_19_O_9_	Scopolin	7.5
